# Dermal Sinus Tract of Lumbosacral Spine in Children: Patterns on Magnetic Resonance Imaging and Scoring System

**DOI:** 10.7759/cureus.1906

**Published:** 2017-12-04

**Authors:** Jessica Lane, Mark Dias, Mark Iantosca, Thomas Zacharia, R. Shane Tubbs, Elias Rizk

**Affiliations:** 1 Department of Neurosurgery, Penn State Milton S Hershey Medical Center, Hershey, USA; 2 Department of Diagnostic Radiology, Penn State Milton S Hershey Medical Center, Hershey, USA; 3 Neurosurgery, Seattle Science Foundation, Seattle, USA

**Keywords:** dermal sinus tract (dst), magnetic resonance imaging (mri), lumbar spine, sacral dimple

## Abstract

Purpose

Dermal sinus tract (DST) is a challenging clinical diagnosis in children. The purpose of our study was to analyze the added value of magnetic resonance imaging (MRI) in the diagnosis of DST involving the lumbosacral spine. We also sought to establish an MRI-based scoring system to simplify the diagnosis of DST.

Methods

MRI images of 20 patients with clinically suspected DST were retrospectively assessed by two neuroradiologists blinded to the surgical results. The MRI studies were performed from July 2003 to July 2013. Institutional Review Board (IRB) approval was obtained. All MRI studies were assessed with respect to five imaging signs: A) visualization on both sagittal and axial images, B) dural penetration, C) associated tethered cord, D) presence of tumor or inflammation, and E) attachment to conus medullaris. The frequency of each imaging sign in the study population was calculated. For the 20 patients who underwent surgery, sensitivity and specificity of each neuroradiologist’s interpretation of the MRI data were calculated using operative findings as the gold standard.

Results

Twelve of the 20 had confirmed DSTs. The incidences of the five imaging signs were as follows: A) visualization on both sagittal and axial images (12/12, 100%), B) dural penetration (10/12, 83.3%), C) associated tethered cord (7/12, 58.3%), D) presence of tumor or inflammation (4/12, 33.3%), and E) attachment to the conus medullaris (4/12, 33.3%). The best combination of findings predictive of DST was simply the appearance of DST on both axial and sagittal imaging, which resulted in a sensitivity of 100% and a specificity of 75-100%.

Conclusion

Visualization of DST on both axial and sagittal imaging is the best marker for pathology proven DST. Using a higher threshold score on the five-point scoring system that we proposed did not increase sensitivity or specificity in the diagnosis of DST; however, it may still prove clinically helpful in standardizing reporting leading to a more accurate and detailed assessment.

## Introduction

Dermal sinus tract (DST) is a rare entity with an incidence of one in 2,500 live births [1]. Embryologically, this rare occult spinal dysraphism is thought to result from the failure of the neuroectoderm to separate completely from the surface ectoderm and its dermal components during the third to the eighth week of gestation [2]. DST is a multilayered epithelium-lined tract that can extend from the skin or subcutaneous tissues to terminate anywhere in between or up to the spinal cord or filum terminale [1]. The areas in which the termination points of DST lie are dorsal to the spinal elements (6-7%), extradural space (10-20%), and intradural space (58-60%) [3]. Specific sites can be the dural surface, intradural surface, filum terminale, lipoma, intradural abscess, spinal cord, or epidural abscess [4].

Clinical features reported in the literature include skin lesions (dimple above gluteal cleft, hypertrichosis, sinus ostium, abnormal pigmentation, subcutaneous lipoma, telangiectasia, and cerebrospinal fluid leak), infections (isolated or recurrent meningitis and abscess), neurological signs (motor weakness, urological problems, and pain), and bony deformity/anomaly [4]. Benign sacral dimple and pilonidal sinus are clinical mimics of DST. The mimics are confined to the gluteal cleft while a DST originates above the cleft and can communicate with the spinal canal and dura mater [2, 5].

To differentiate these clinical mimics and to exclude DST in patients with sacral dimples, magnetic resonance imaging (MRI) is frequently indicated [5]. Detection and characterization of the lesion, localization of its extent, and description of associated pathology and complications are all achieved with high accuracy by MRI in cases of DST [6]. Higher resolution scanners and new imaging sequences have increased the accuracy of diagnosis and early diagnosis over the years [1-2]. The primary objective of this study is to define the value of MRI in the diagnosis of DST. The second objective is to derive an MRI scoring system to establish a simplified approach to the diagnosis of DST.

DST is a rare disorder. Major clinical presentations include skin lesions, infections, and neurological signs [4]. DST is treated by resection of the tract to prevent complications such as meningitis and to reduce neurological morbidity due to cord tethering. Repeat surgical exploration is necessary in patients where DST is associated with other intradural or extradural pathologies and their complications [4]. Magnetic resonance imaging is frequently indicated to diagnose and characterize DST and to plan treatment [5-6]. DST, though rare, can present anywhere along the neuraxis from the nasal ridge to the distal spine. It is most common in the lumbar region [7]. MRI manifestations vary in complexity from simple lesions terminating in an extradural location to complex lesions communicating with the spinal cord with associated abscesses or masses such as dermoids [4, 6].

## Materials and methods

Study design and setting

The study was approved by the institutional review board (IRB), and Health Insurance Portability and Accountability Act (HIPAA) compliance was maintained throughout. MR images of 20 patients from July 2003 to July 2013 with clinically suspected dermal sinus tract (DST) were retrospectively analyzed by two neuroradiologists. All 20 of these patients had undergone surgery in the area of the suspected DST, although some for purposes other than DST resection. These neuroradiologists were blinded to the presence or absence of DST on pathology.

Imaging technique

MRI of the lumbar spine was performed using a 1.5T Intera System Machine (Philips Medical System, Best, The Netherlands) using a spine coil (Philips Health System, Best, The Netherlands). In supine position, T1 and T2-weighted images were acquired in both the axial and sagittal planes. An FOV of 32 cm, matrix 512 × 512, and section thickness of 3 mm were used for sagittal views; an FOV of 16 cm, matrix 320 × 320, and section thickness of 4 mm were used for axial images. The MR images were sagittal T1-weighted spin-echo (TR/TE = 500–600/10–17 ms), T2-weighted (TR/TE, 2750/110 ms), axial T2-weighted spin-echo (TR/TE, 4000-5000/120 ms), and T1-weighted spin-echo (TR/TE, 500-600/10–15 ms).

Imaging analysis and definition of scoring criteria/imaging sign

The imaging studies were reviewed by two subspecialty-certified neuroradiologists with more than 10 years experience. The location and extent of DST, dural penetration, association with tethered cord, coexisting masses, syringomelia, and signs of inflammation were determined. All cases were assessed for five scoring criteria/imaging signs to establish a five-point scoring system.

Definition of scoring criteria/imaging sign

1. Visualization on both sagittal and axial images: the DST tract was clearly identified on both axial and sagittal T1 and T2-weighted images. The maximum tract diameter was measured on both axial and sagittal sequences.

2. Dural penetration: the tract could be traced from the skin extending through the bone, epidural space and dura with entry into the thecal sac.

3. Associated tethered cord: the tip of the conus medullaris was seen below mid L2 level.

4. Presence of mass, inflammation, or syrinx: any mass such as lipoma or dermoid. Inflammation was defined by findings of abscess, infected collections, or imaging findings of meningitis. Syrinx was any T2 bright enlargement of the central canal >1 mm.

5. Attachment to conus medullaris: DST could be traced to attachment to the conus medullaris.

Statistical analysis

The sensitivity and specificity of interpretation of MRI data by each neuroradiologist were calculated using multiple scoring thresholds for positive diagnosis.

## Results

Patient population

This cohort of 20 patients reviewed were part of a larger cohort of 40 patients with clinically suspected DST. All patients had clinical evidence of a low lumbar dimple or defect above the gluteal cleft, and MRI was clinically recommended to establish the diagnosis of DST and associated pathological conditions. The 20 patients in this study had pathology proving presence or absence of DST.

MRI findings

Among the 20 patients, DST was identified on imaging in 12 and 14 patients by neuroradiologist 1 and 2, respectively. All of neuroradiologist 1’s positive findings were also identified by neuroradiologist 2. Surgery and pathology confirmed the diagnosis of DST in 12 patients: all of those jointly identified by both radiologists, but not the two patients identified only by neuroradiologist 2. All 12 patients with pathology-proven DST had a single DST in the lumbosacral region. The spinal cord levels affected were from L4 to S3. Among the confirmed cases, 10 had midline skin dimpling while two had skin dimpling in a right paramedian lumbosacral location. Both neuroradiologists visualized the tract on both sagittal and axial images in all confirmed cases of DST.

Incidences of imaging signs/criteria of the five-point score were as follows (Table 1):

**Table 1:** Incidences of imaging signs and criteria

	Imaging signs/criteria	Incidence in DST population (neuroradiologist 1)	Incidence in DST population (neuroradiologist 2)
A.	Visualization on axial and sagittal images	12/12,100%	12/12,100%
B.	Dural penetration	10/12, 83.3%	9/12, 75%
C.	Associated tethered cord or thick filum	7/12, 58.3%	7/12, 58.3%
D.	Presence of mass, abscess or syrinx	5/12, 41.7%	5/12, 41.7%
E.	Attachment to conus medullaris	4/12, 33.3 %	4/12, 33.3 %

Twelve patients were confirmed to have DST by surgery and pathological analysis. All 12 cases had one or more imaging signs as listed above along with clinical features of DST. All were single and situated in the midline, except for two, which were paramedian. The eight patients without DST identified on MRI had undergone surgery for indications other than DST.

Using a threshold score of 1 provided a sensitivity of 100% and specificity of 75% for both neuroradiologists. Just using the criteria of seeing the DST on both axial and sagittal imaging increased the specificity of neuroradiologist 1 to 100% without affecting sensitivity and specificity for neuroradiologist 2. Increasing our threshold to two points on the MRI scale lowered the sensitivity to 92% for both radiologists, leaving specificity for radiologist 1 at 100% and radiologist 2 at 75%. Using criteria A (visualization on axial and sagittal imaging) also yielded a positive predictive value of 100% for neuroradiologist 1 and 86% for neuroradiologist 2, and negative predictive values of 100% for both readers.

## Discussion

DST, though rare, can present anywhere along the neuraxis from calvaria to coccyx. MRI manifestations vary in complexity from simple lesions terminating in an extradural location to complex lesions communicating with the spinal cord with associated infections, abscesses, or masses such as dermoids [4]. While MRI can be an excellent tool for diagnosis of DST, there are cases in which the tract has low signal intensity in MRI, so the imaging can still fail to show DST in clinically suspected and surgically/pathologically confirmed cases. Hence, we need to keep the pathologies that are commonly associated with DST in mind and look at the images with variable window settings so as not to overlook it or its associated pathologies [8]. Additionally, there can be vascular channels that can be mistaken for DST especially in cases with other supporting findings such as dermoid or abscess. In our study, when the MRI score is at least 1/5 in clinically suspected cases, there is a high chance that the pathology can be diagnosed as DST by surgery and pathology. In fact, simply identifying the DST on both axial and sagittal imaging yielded the test with the highest sensitivity and specificity. The requirement of additional supportive findings or higher MRI score does not increase sensitivity or specificity. Despite this fact, evaluating images using our five-point MRI scale may have clinical importance by encouraging readers to check for potentially harmful associated findings.

Clinical implications

Treatment of DST is by surgical exploration up to the termination point of the DST and resection of the tract to prevent complications such as meningitis and abscess due to the seeding of pathogens into the tract and to reduce neurological morbidity due to cord tethering, cord compression, or other spine deformities/anomalies. Repeat surgical exploration may be necessary in patients where DST is associated with other intra- and extradural pathologies and such associated complications as infection, abscess formation and cord tethering [3].

Limitations

Our study investigated a small patient population. DST being a rare entity, it was difficult to enroll patients who met the inclusion criteria. The retrospective nature of the study probably resulted in an inherent bias towards suspecting a diagnosis of DST in the patients who were analyzed. Additionally, we only reviewed patients who had operative pathology as we consider this to be the gold standard of diagnosis. This led to a mixed pool of patients including those with very high suspicion for DST or other pathology requiring surgery. Our study population did not include patients for whom clinical or imaging findings were not so convincing as to justify surgery. These same neuroradiologists reviewed the MRIs for 20 additional patients with clinical concern for DST who did not undergo surgery. DST was identified in one of these patients by a single neuroradiologist. These patients were followed and did not develop symptoms of DST; however, these patients were not included in the present study due to lack of definitive diagnosis. A multi-institutional prospective MRI analysis of this nature can be difficult to conduct because of the difficulty of enrolling newborns and very young children who present clinically with this problem.

## Conclusions

Diagnosing dermal sinus tracts can be difficult. The best single predictor of DST in our population of patients was visualization on both sagittal and axial imaging. We proposed a five-point scoring system with five parameters that can be used to diagnose DST on MRI. While higher score thresholds did not increase the sensitivity and specificity of the test, they do outline clinically important findings that should be specifically examined when reviewing films in a patient with clinically suspected DST. Potentially, this scoring system could have clinical implications for detailed delineation of associated pathologies including infection, intradural mass, and syringomelia for more standardized and consistent image interpretation.
